# Transcriptomic Dynamics and ECM Remodeling During Postnatal Testicular Maturation in the Tianzhu White Yak

**DOI:** 10.3390/ani16182853

**Published:** 2026-09-10

**Authors:** Yu Shi, Bingang Shi, Youpeng Qi, Changze Cui, Meixian Zhang, Zhihao Luo, Yuwei Ma, Jiang Hu

**Affiliations:** Gansu Key Laboratory of Herbivorous Animal Biotechnology, College of Animal Science and Technology, Gansu Agricultural University, Lanzhou 730070, China

**Keywords:** yak, testis, spermatogenesis, puberty, transcriptome, WGCNA, RNA-seq, extracellular matrix

## Abstract

The molecular mechanisms underlying testicular development in the yak remain poorly understood. In this study, RNA sequencing was performed on testicular tissues from Tianzhu white yaks at three postnatal developmental stages: 0.5 (prepubertal), 2.5 (pubertal), and 4.5 (adult) years, with six animals in each group. Differential expression analysis was integrated with weighted gene co-expression network analysis (WGCNA) to characterize transcriptomic changes associated with testicular maturation. A total of 9646 differentially expressed genes (DEGs) were identified between the 0.5- and 2.5-year groups, whereas only 126 DEGs were detected between the 2.5- and 4.5-year groups, indicating that the transition to puberty represents the most dynamic phase of transcriptional reprogramming during testicular development. Functional enrichment analysis revealed that focal adhesion and extracellular matrix–receptor interaction were the most significantly enriched Kyoto Encyclopedia of Genes and Genomes pathways, suggesting a potential role for extracellular matrix (ECM) remodeling in the establishment of the blood–testis barrier and testicular maturation. WGCNA identified seven age-associated co-expression modules. The turquoise module showed the strongest positive correlation with developmental stage and was significantly enriched for genes involved in spermatid differentiation and maturation, including *SPATA6*, *SPMIP11*, *TNP1*, and *ODF1*. Collectively, these findings provide a comprehensive transcriptomic characterization of yak testicular development.

## Simple Summary

Yaks (*Bos grunniens*) are vital to the livelihoods of pastoral communities on the Qinghai–Tibetan Plateau; however, their reproductive performance is constrained by late sexual maturity, strong seasonal breeding patterns, and low conception rates. A better understanding of the molecular mechanisms underlying testicular development may facilitate the development of strategies to improve yak fertility. In this study, we analyzed gene expression profiles in the testes of Tianzhu white yaks at three key developmental stages: prepuberty (0.5 years), puberty (2.5 years), and adulthood (4.5 years). The most pronounced transcriptional changes occurred during the transition from prepuberty to puberty, involving nearly 10,000 genes, whereas comparatively few genes changed between puberty and adulthood. Gene network analysis identified groups of co-regulated genes associated with spermatogenesis, and functional analyses revealed that remodeling of the cellular scaffold (extracellular matrix) may play an important role during this critical developmental period. These findings provide a comprehensive molecular framework for understanding testicular maturation in yaks and identify biological pathways that may serve as potential targets for improving reproductive performance.

## 1. Introduction

The yak is the principal livestock species on the Qinghai–Tibetan Plateau, providing meat, milk, fibre, and draft power for millions of pastoralists [1,2]. The Tianzhu white yak, a distinctive white-coated breed native to the Tianzhu Tibetan Autonomous County in Gansu Province, China, is renowned for its adaptation to the harsh high-altitude environment (2400–4000 m) of the Qinghai–Tibetan Plateau [3,4]. With a current population of approximately 152,800 individuals (2025), this breed is of substantial cultural and economic importance to local pastoral communities [4]. Despite its economic importance, reproductive efficiency in yaks remains low because of late sexual maturity, strong breeding seasonality, and relatively low conception rates compared with other domestic bovids [5,6,7,8]. Understanding the molecular mechanisms regulating testicular development and spermatogenesis is therefore important for both reproductive biology and breeding improvement [9].

Testicular development and spermatogenesis are tightly regulated processes that require coordinated gene expression across multiple cell types [10,11,12,13,14]. In mammals, postnatal testicular maturation progresses through distinct developmental stages, including a prepubertal phase characterized by Sertoli cell proliferation and spermatogonial stem cell self-renewal [15], a pubertal phase marked by the onset of meiosis, blood–testis barrier (BTB) formation, and the appearance of haploid spermatids, and an adult phase characterized by sustained spermatogenesis [11,16]. With the development of RNA sequencing (RNA-seq), transcriptomic analyses of testicular development have been reported in cattle (*Bos taurus*) [13,16], sheep (*Ovis aries*) [17,18], pigs (*Sus scrofa*) [19], chickens (*Gallus gallus*) [20], and goats (*Capra hircus*) [14,21,22], revealing both conserved and species-specific regulatory mechanisms.

In contrast, the molecular characterization of testicular development in yaks remains limited. Whole-transcriptome studies have characterized mRNAs, lncRNAs, circRNAs, and miRNAs during yak testicular development [23,24,25,26]. In addition, molecular approaches have also begun to clarify other aspects of yak reproduction, including inflammatory regulation in the endometrium [27]. Full-length transcriptome sequencing (Iso-Seq) has been used to identify candidate genes associated with spermatogenesis and characterize developmental changes in the yak testis [28,29]. Single-cell RNA-seq studies have primarily focused on cattle–yak hybrids to investigate male sterility [22,30], while analyses of purebred yaks have been restricted largely to adult testes at a single developmental stage [28]. However, the integration of bulk RNA-seq with weighted gene co-expression network analysis (WGCNA) across multiple developmental stages within a single yak breed remains limited. Moreover, extracellular matrix (ECM) and focal adhesion signaling pathways, which play important roles in blood–testis barrier (BTB) dynamics and germ cell migration [31,32], have not been systematically investigated during yak testicular development. WGCNA enables the identification of co-expressed gene modules associated with biological traits and provides a systems-level perspective beyond conventional differential expression analyses [33]. Whereas conventional differential expression analysis identifies individual genes whose expression changes between developmental stages, WGCNA reveals co-expression modules systematically associated with developmental age and prioritizes hub genes that occupy central positions within these modules, thereby uncovering regulatory genes that may not be the most strongly differentially expressed but may still play important roles in coordinating testicular maturation. By applying WGCNA to a single breed sampled across three postnatal developmental stages with six biological replicates per stage, this study provides a network-level framework linking co-expression modules and hub genes to the transcriptional reprogramming of the pubertal transition, including the coordinated remodeling of the ECM and cell adhesion signaling.

Therefore, in this study, we performed RNA sequencing of testicular tissues from Tianzhu white yaks at 0.5 (prepubertal), 2.5 (pubertal), and 4.5 (adult) years of age, with six biological replicates per stage. By integrating differential expression analysis with WGCNA, we aimed to (1) characterize transcriptomic changes during testicular maturation, (2) identify age-associated co-expression modules and key hub genes, and (3) investigate the biological pathways underlying the pubertal transition, with particular emphasis on ECM-related signaling.

## 2. Materials and Methods

### 2.1. Animals and Sample Collection

All experimental procedures were approved by the Institutional Animal Care and Use Committee of Gansu Agricultural University (approval number: GSAU-Eth-AST-2023-014). Eighteen healthy male Tianzhu white yaks (*Bos grunniens*) were obtained from a commercial farm in Tianzhu Tibetan Autonomous County, Gansu Province, China, during the cold season. Based on age, animals were assigned to three developmental groups: 0.5 years (prepubertal, *n* = 6), 2.5 years (pubertal, *n* = 6), and 4.5 years (adult, *n* = 6). Six biological replicates were included per group, consistent with recommendations for RNA-seq differential expression studies [34]. The body weights of the animals were 39.33 ± 2.62 kg (0.5 years), 113.50 ± 5.38 kg (2.5 years), and 158.40 ± 7.13 kg (4.5 years), as reported in our previous study [35]. Information regarding feeding management, diet composition, and housing conditions has been reported previously [35]. Throughout the study, all animals were maintained under identical husbandry conditions with ad libitum access to water. Experimental procedures and reporting complied with the ARRIVE 2.0 guidelines [36]. To minimize observer bias, histological evaluations were conducted by a trained investigator blinded to group allocation.

Animals were euthanized by electrical stunning followed by exsanguination in accordance with approved animal welfare protocols. Immediately after slaughter, testicular tissues were excised and processed for downstream analyses. For each animal, the testicular parenchyma was divided into two portions: one portion was snap-frozen in liquid nitrogen and stored at −80 °C for RNA extraction, whereas the other was fixed in 4% paraformaldehyde for histological examination.

### 2.2. RNA Extraction, Library Construction, and Sequencing

Total RNA was extracted from testicular tissues using TRIzol reagent (Invitrogen, Carlsbad, CA, USA) according to the manufacturer’s instructions. RNA integrity was assessed using an Agilent 2100 Bioanalyzer (Agilent Technologies, Santa Clara, CA, USA), and all samples exhibited RNA integrity numbers (RINs) ≥ 7.0, meeting the quality requirements for RNA sequencing [35]. RNA concentration and purity were determined using a NanoDrop 2000 spectrophotometer (Thermo Fisher Scientific, Waltham, MA, USA). Sequencing libraries were prepared using the TruSeq Stranded mRNA Library Prep Kit (Illumina, San Diego, CA, USA). Briefly, polyadenylated mRNA was enriched from total RNA using oligo(dT)-coupled magnetic beads and subsequently fragmented before cDNA synthesis. Following end repair, A-tailing, adapter ligation, and PCR amplification, the libraries were sequenced on an Illumina NovaSeq X Plus platform (Illumina, San Diego, CA, USA) by Shanghai Majorbio Bio-pharm Technology Co., Ltd. (Shanghai, China), generating 150-bp paired-end reads.

### 2.3. Read Filtering and Mapping

Raw sequencing reads were processed using fastp (v0.23.4) (https://github.com/OpenGene/fastp, accessed on 8 September 2026), a widely used tool for quality control and adapter trimming of high-throughput sequencing data, to remove adapter sequences and low-quality reads [37]. Reads with quality scores below Q20 or containing more than 5% ambiguous nucleotides (N) were discarded. The resulting clean reads were aligned to the yak reference genome (BosGru_v3.0) using HISAT2 (v2.2.1) with default parameters. Aligned reads were assembled into transcripts using StringTie (v2.2.1) in a reference-guided manner, and transcript abundance was quantified with RSEM (v1.3.3) [38]. Gene expression levels were normalized as transcripts per million (TPM). In total, 21,698 genes were detected across all samples. Following preprocessing on the Majorbio Cloud Platform, genes with low expression and low variability were removed based on a mean expression ≥ 1 and a coefficient of variation ≥ 0.1, resulting in 11,602 genes being retained for subsequent analyses.

### 2.4. Differential Expression Analysis

Differential gene expression analysis was performed using DESeq2 (v1.42.0) [39]. Pairwise comparisons were conducted among the three developmental stages (0.5 vs. 2.5 years, 0.5 vs. 4.5 years, and 2.5 vs. 4.5 years). Genes with an absolute log_2_ fold change (|log_2_FC|) > 1 and a Benjamini–Hochberg adjusted *p*-value < 0.05 were considered differentially expressed genes (DEGs). All 18 samples were retained for RNA-seq analysis; quality metrics for each sample (Q20 ≥ 99.2% and unique mapping rate ≥ 78.6%) met or exceeded standard thresholds. Principal component analysis (PCA) and sample-to-sample correlation heatmaps were generated to evaluate global transcriptomic variation and assess sample clustering across developmental stages.

### 2.5. Functional Enrichment Analysis

Gene Ontology (GO) enrichment analysis was performed using Goatools (v1.4.4), and Kyoto Encyclopedia of Genes and Genomes (KEGG) pathway enrichment analysis was conducted using the SciPy package (v1.0.0) on Python 2.7, all through the Majorbio Cloud Platform. Enrichment significance was evaluated using Fisher’s exact test, followed by Benjamini–Hochberg correction for multiple testing. The background gene set for enrichment analysis consisted of all 21,698 expressed genes detected in the testicular transcriptome. GO terms were classified into the biological process (BP), cellular component (CC), and molecular function (MF) categories. GO terms and KEGG pathways with adjusted *p*-values < 0.05 were considered significantly enriched. All analyses were performed using the Majorbio Cloud Platform (Shanghai Majorbio Bio-pharm Technology Co., Ltd., Shanghai, China). Complete GO and KEGG enrichment results for all comparisons are provided in Tables S3 and S4.

### 2.6. Weighted Gene Co-Expression Network Analysis (WGCNA)

WGCNA was performed using the WGCNA R package (v1.73) [33,40]. All 11,602 genes that passed the platform preprocessing criteria (mean expression ≥ 1 and coefficient of variation ≥ 0.1) were retained for network construction, and the input matrix was log2(TPM + 1)-transformed. The soft-thresholding power (β) was determined using the pickSoftThreshold function, and a value of 20 was selected as the lowest power at which the scale-free topology fit index (R^2^) reached 0.8667. An unsigned co-expression network was then constructed using the blockwiseModules function with the following parameters: networkType = unsigned, power = 20, minModuleSize = 30, minKMEtoStay = 0.3, mergeCutHeight = 0.25. Module eigengenes, representing the principal expression pattern of each module, were calculated as the first principal component of the corresponding module. Associations between module eigengenes and the three developmental groups (0.5, 2.5, and 4.5 years; *n* = 6 per group) were evaluated using Pearson correlation analysis, with Bonferroni correction applied across 21 tests (7 modules × 3 groups). To address the concern that a soft-thresholding power of 20 may constitute overcorrection, a sensitivity analysis was performed by independently re-running WGCNA at β = 6, 8, 10, 12, 14, 16, 18, and 20 in R; the four core hub genes (*SPATA6*, *SPMIP11*, *TNP1*, and *ODF1*) remained in the turquoise module with |kME| > 0.99 across all β values, confirming the robustness of the module assignment (Table S2). Hub genes were defined as genes exhibiting both high module membership (|kME| > 0.80) and strong age-related significance within a given module.

### 2.7. Histological Analysis

Testicular tissues fixed in 4% paraformaldehyde were dehydrated through a graded ethanol series, cleared in xylene, embedded in paraffin, and sectioned at a thickness of 5 μm. The sections were mounted on glass slides and stained with hematoxylin and eosin (H&E) as previously described [41]. Histological features were examined using a light microscope equipped with a digital imaging system to evaluate age-related changes in testicular architecture and seminiferous tubule development across the three developmental stages.

### 2.8. RT-qPCR Validation

To validate the RNA-seq results, six differentially expressed genes (*SPATA6*, *TNP1*, *COL4A1*, *COL6A1*, *FN1*, and *ITGAV*) were selected for reverse transcription quantitative PCR (RT-qPCR) quantification. These genes were chosen based on their differential expression across developmental stages and their relevance to testicular maturation and ECM remodeling pathways. Two additional hub genes identified by WGCNA (*ODF1*, |kME| = 0.999; *SPMIP11*, |kME| = 0.997) were excluded from qPCR validation because preliminary primer optimization experiments failed to produce specific amplification products with acceptable melt curves. Total RNA was reverse-transcribed using SweScript All-in-One RT SuperMix for qPCR containing gDNA Remover (Servicebio, Wuhan, China, Cat# G3337). Quantitative PCR was performed on a CFX Connect Real-Time PCR Detection System (Bio-Rad, Hercules, CA, USA) using 2× Universal Blue SYBR Green qPCR Master Mix (Servicebio, Cat# G3326). Each 15 µL reaction contained 7.5 µL SYBR Green Master Mix, 1.5 µL gene-specific primers (2.5 µM each), 2.0 µL cDNA template, and 4.0 µL nuclease-free water. The thermal cycling protocol consisted of an initial denaturation at 95 °C for 30 s, followed by 40 cycles of 95 °C for 15 s and 60 °C for 30 s. Melt curve analysis was performed by ramping from 65 °C to 95 °C at 0.5 °C increments. All reactions were performed in triplicate. β-actin was used as the endogenous reference gene, and relative expression levels were calculated using the 2^−ΔΔCt^ method. Primer sequences and amplification conditions are provided in Table S5.

## 3. Results

### 3.1. Testicular Maturation Progresses Markedly During Puberty

**Figure 1:**
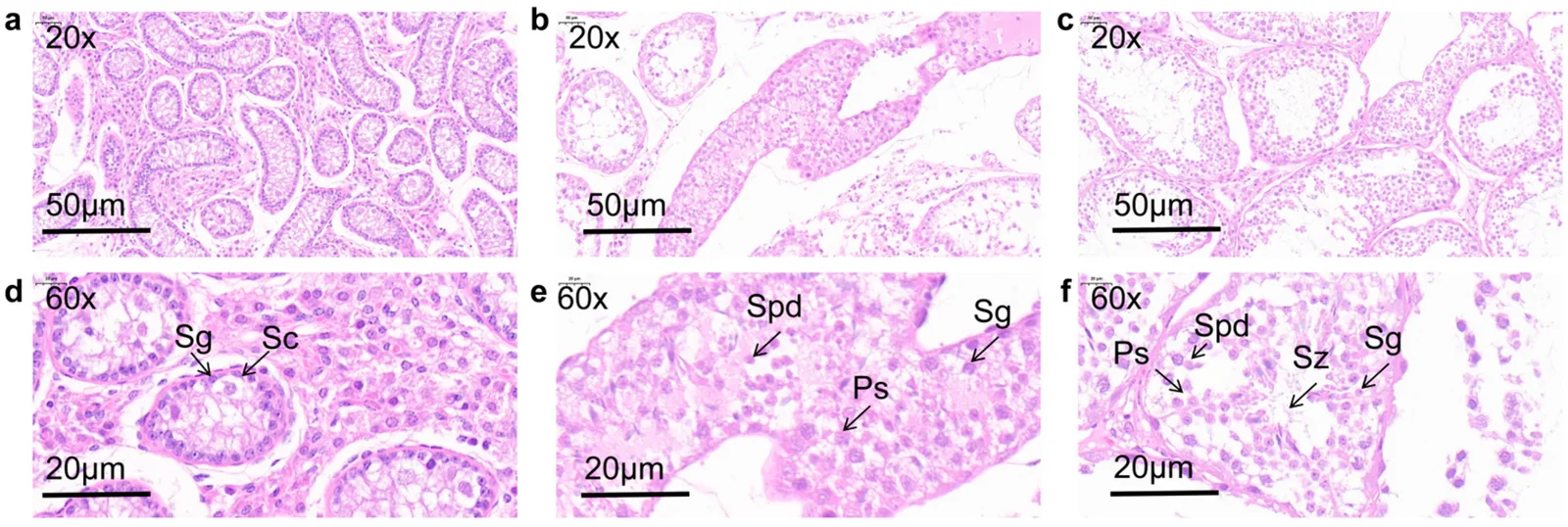
Hematoxylin and eosin staining of yak testicular sections across three postnatal developmental stages. (**a**) Pre-pubertal (0.5 years). (**b**) Pubertal (2.5 years). (**c**) Adult (4.5 years). (**d**–**f**) Higher-magnification views of the boxed regions in (**a**–**c**), respectively, showing the progressive maturation of testicular histology across the three developmental stages.

H&E-stained testicular sections revealed progressive morphological maturation across the three developmental stages (Figure 1). At 0.5 years, seminiferous tubules were relatively small and contained predominantly Sertoli cells and spermatogonia, with few spermatocytes and no detectable spermatids, consistent with a prepubertal state (Figure 1a). By 2.5 years, the seminiferous tubule diameter had increased substantially, and the seminiferous epithelium contained a complete spectrum of germ cells, including spermatogonia, primary and secondary spermatocytes, and abundant elongating spermatids, indicating the onset of active spermatogenesis (Figure 1b). At 4.5 years, the testes exhibited a fully mature morphology characterized by well-organized spermatogenic layers and abundant spermatozoa within the tubular lumen (Figure 1c). Collectively, these histological observations confirm that the sampled animals represent distinct prepubertal (0.5 years), pubertal (2.5 years), and adult (4.5 years) stages of testicular development.

### 3.2. High-Quality Transcriptomes Were Generated Across Developmental Stages

To characterize the transcriptomic changes accompanying testicular maturation, RNA sequencing was performed on testicular tissues from all 18 animals. Following quality filtering, an average of 39.1 million clean reads per sample was obtained. All libraries exhibited high sequencing quality, with Q20 scores exceeding 99% and Q30 scores exceeding 96% (Table S1). Alignment to the yak reference genome (BosGru_v3.0) yielded an average mapping rate of 93.1%, including approximately 79.5% uniquely mapped reads (Table 1). In total, 21,698 expressed genes were detected, representing approximately 87.0% of the 24,945 annotated protein-coding genes in the reference genome. Following preprocessing on the Majorbio Cloud Platform (mean expression ≥ 1 and coefficient of variation ≥ 0.1), 11,602 genes were retained for differential expression and WGCNA analyses (Table 1).

**Figure 2:**
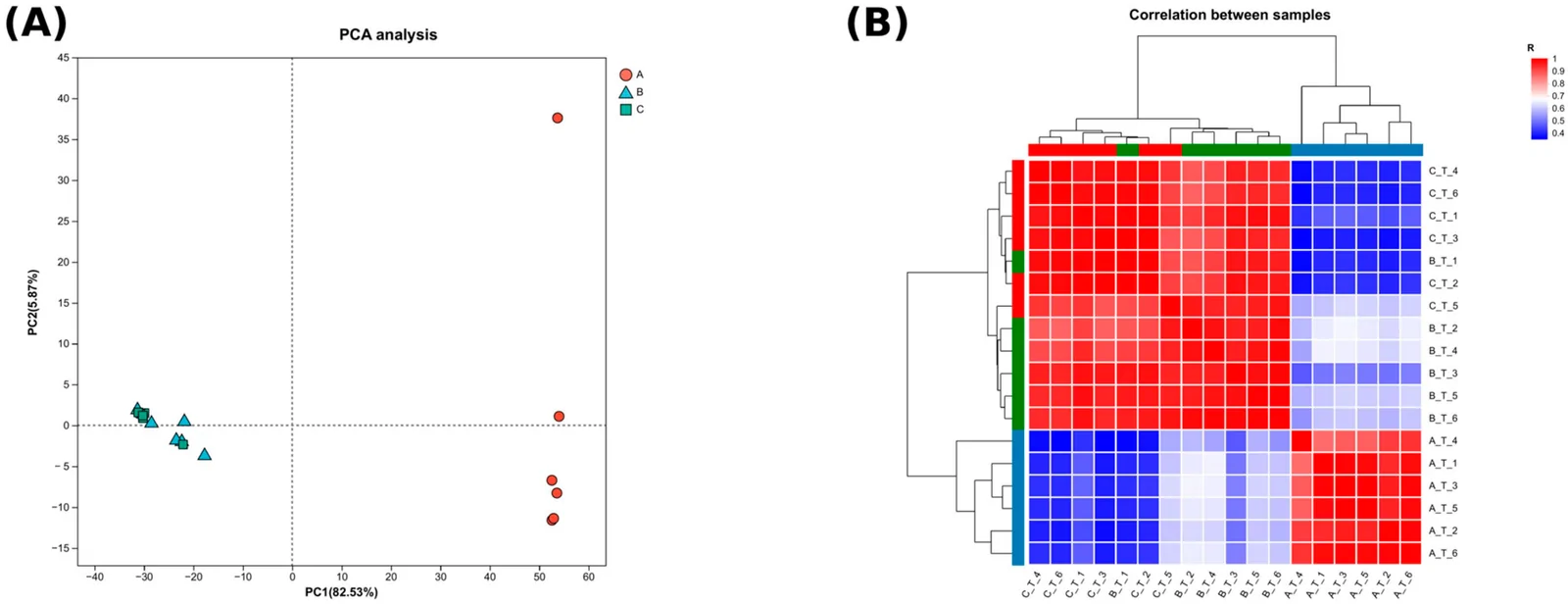
Developmental stage-dependent clustering of yak testicular transcriptomes. (**A**) Principal component analysis (PCA) of testicular samples from the three age groups; red, blue, and green dots represent the 0.5-, 2.5-, and 4.5-year groups, respectively. PC1 and PC2 explained 82.5% and 5.9% of the total variance, respectively. (**B**) Sample-to-sample Pearson correlation heatmap; the color bar indicates correlation strength (blue, low; red, high), and the sample group annotation (blue, green, and red representing the 0.5-, 2.5-, and 4.5-year groups, respectively) is displayed adjacent to the heatmap.

PCA revealed clear separation of samples according to developmental stage (Figure 2A). PC1, which explained 82.5% of the total variance, distinctly separated the 0.5-year group from the 2.5- and 4.5-year groups, whereas PC2 (5.9% of the variance) further discriminated between the 2.5- and 4.5-year groups. Consistent with the PCA results, sample-to-sample correlation analysis showed strong within-group reproducibility, with Pearson correlation coefficients exceeding 0.92 in all age groups (Figure 2B). Notably, the 2.5- and 4.5-year groups exhibited greater transcriptional similarity to each other than to the 0.5-year group, indicating that the most substantial transcriptomic changes occur before or during the pubertal transition. These findings are consistent with the histological evidence of extensive testicular maturation between 0.5 and 2.5 years of age.

### 3.3. Pubertal Transition Drives Extensive Transcriptomic Reprogramming

**Figure 3:**
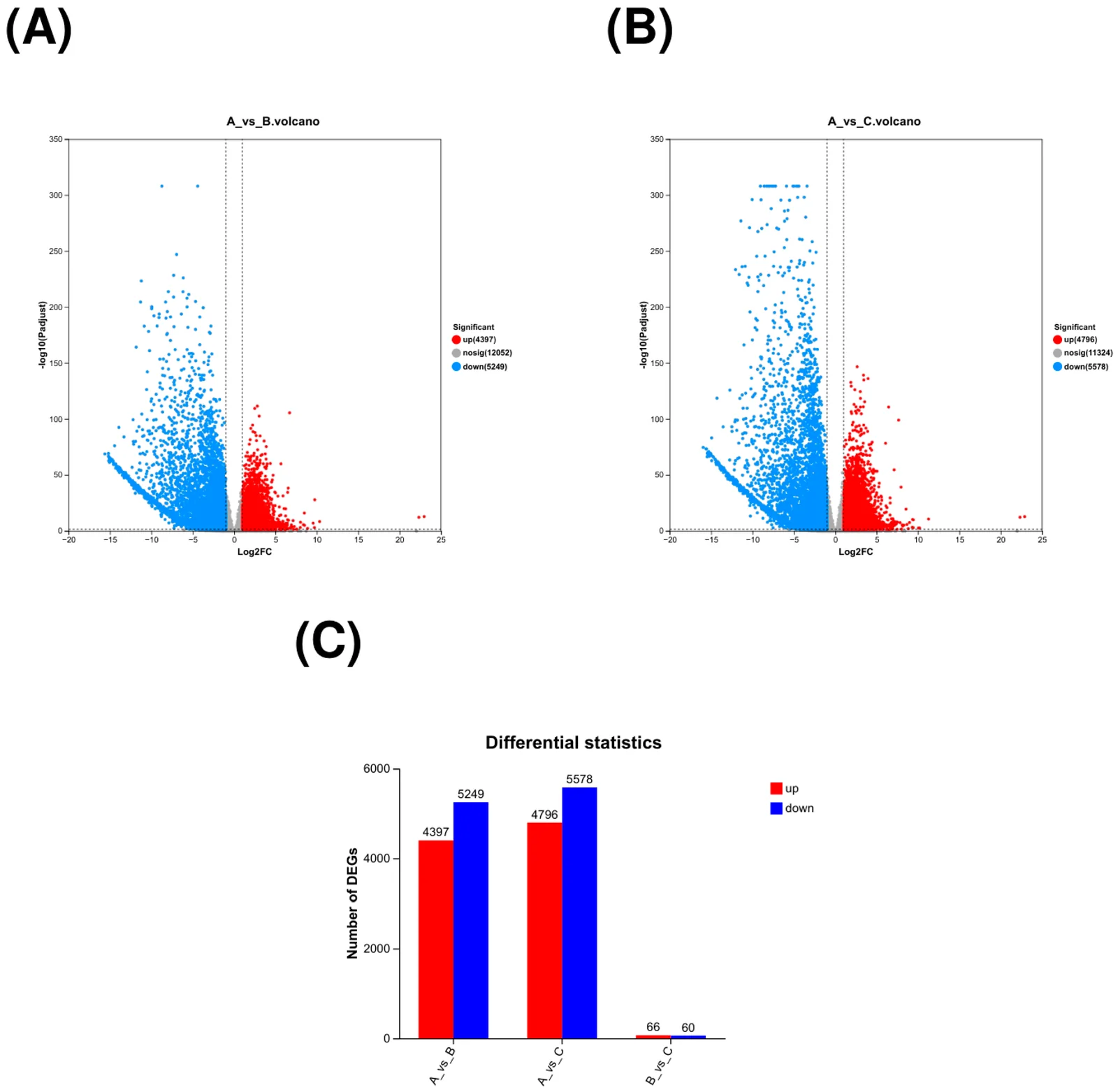
Differential gene expression across developmental stages of yak testicular maturation. Volcano plots showing DEGs (|log_2_FC| > 1, adjusted *p* < 0.05) in the comparisons of (**A**) 0.5 vs. 2.5 years (9646 DEGs), (**B**) 0.5 vs. 4.5 years (10,374 DEGs), and (**C**) 2.5 vs. 4.5 years (126 DEGs). Red and blue dots represent up-regulated and down-regulated genes, respectively. The dashed lines indicate the thresholds of |log2FC| = 1 and adjusted *p* = 0.05.

Pairwise differential expression analysis revealed substantial differences in the extent of transcriptional change across developmental stages. A total of 9646 DEGs (log_2_FC > 1, adjusted *p* < 0.05) were identified between the 0.5- and 2.5-year groups, including 5249 up-regulated and 4397 down-regulated genes in the 2.5-year group (Table 2; Figure 3A). Similarly, 10,374 DEGs were detected between the 0.5- and 4.5-year groups, comprising 5578 up-regulated and 4796 down-regulated genes in the adult group (Figure 3B). In contrast, only 126 DEGs (60 up-regulated and 66 down-regulated) were identified between the 2.5- and 4.5-year groups (Figure 3C). These results indicate that the most extensive transcriptomic reprogramming occurs before or during puberty, whereas relatively few gene expression changes accompany the transition from puberty to adulthood. Complete DEG lists are provided in Table S1.

The DEGs, GO enrichment, and KEGG pathway results for the puberty-to-adult comparison are provided in Figures S1–S4.

### 3.4. ECM Remodeling and Spermatogenesis Pathways Characterize the Pubertal Transition

**Figure 4:**
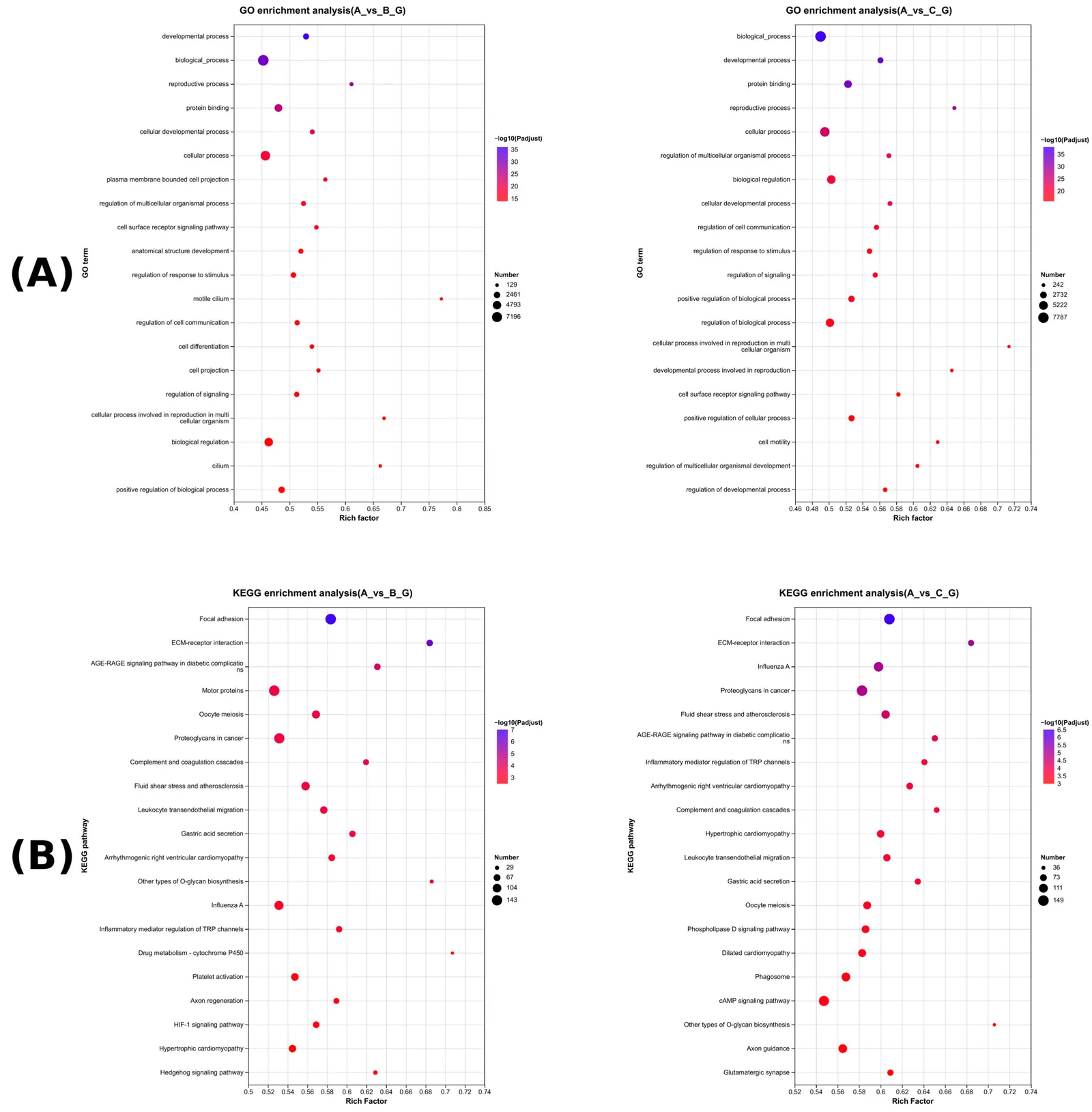
Functional enrichment analysis of DEGs identified in the prepuberty-to-puberty comparison. (**A**) GO enrichment bubble plot showing significantly enriched biological process (BP), cellular component (CC), and molecular function (MF) terms for DEGs between the 0.5- and 2.5-year groups. Bubble size represents gene count, and color intensity indicates the adjusted *p*-value. (**B**) KEGG pathway enrichment bubble plots for the 0.5- versus 2.5-year (**left**) and 0.5- versus 4.5-year (**right**) comparisons, showing the top enriched pathways, including focal adhesion and ECM–receptor interaction as the most significant pathways in both comparisons. Colour gradient indicates adjusted *p*-value, and bubble size represents the number of DEGs in each pathway.

**Figure 5:**
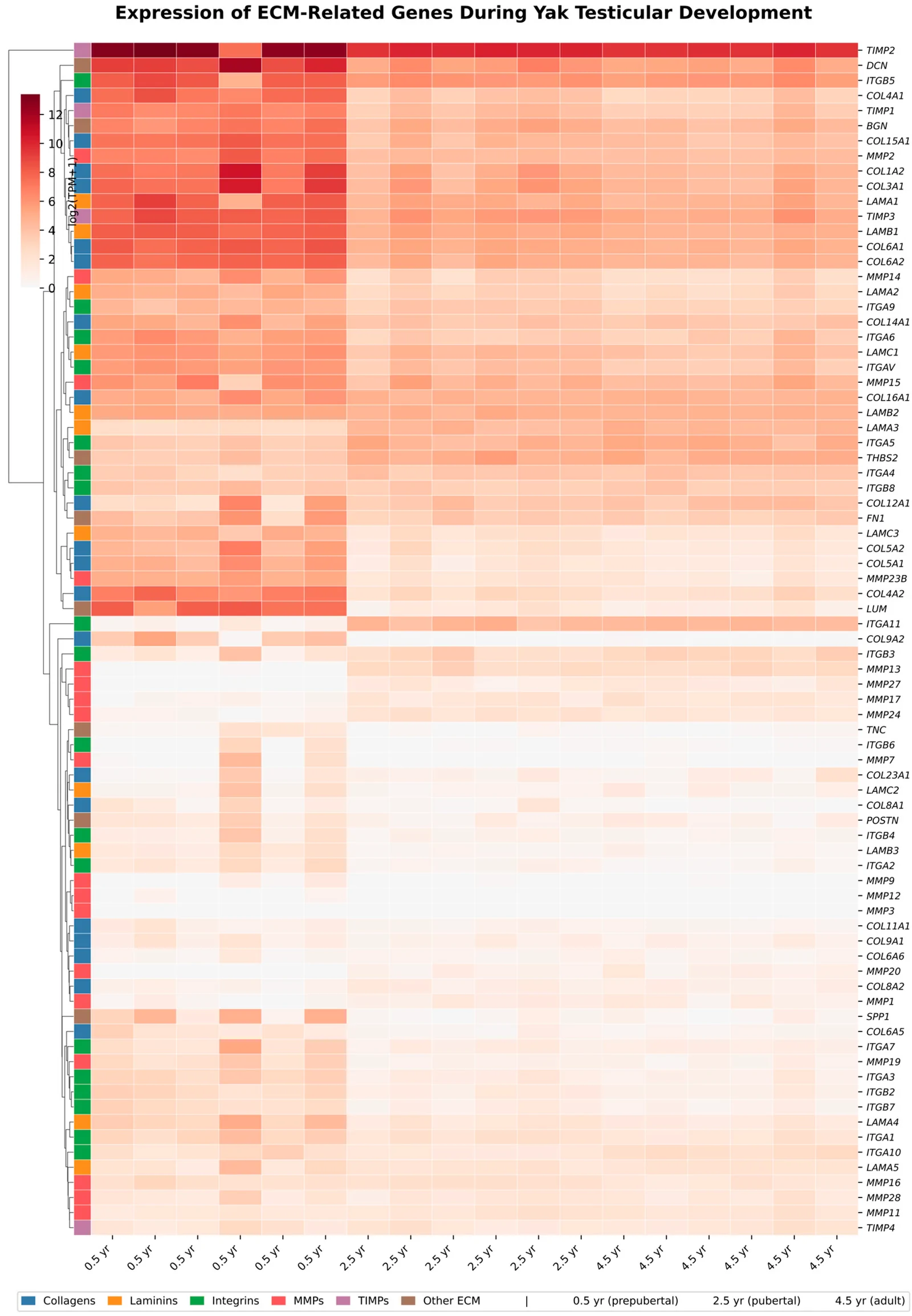
Heatmap of 60 ECM-related genes across the three developmental stages of yak testicular development. The heatmap shows expression patterns of collagens, laminins, integrins, matrix metalloproteinases (MMPs), tissue inhibitors of metalloproteinases (TIMPs), and other ECM components. Each column represents one of the 18 samples (n = 6 per developmental stage), and each row represents one gene. The color scale (white to dark red) represents normalized expression levels (z-score). The left annotation bar indicates the gene category. The developmental stage of each sample is labeled below the heatmap: 0.5 yr (prepubertal), 2.5 yr (pubertal), and 4.5 yr (adult). The heatmap reveals a coordinated down-regulation of ECM-related genes from the prepubertal to the pubertal and adult stages, consistent with active ECM remodeling during the pubertal transition.

Functional enrichment analysis of DEGs identified biological processes and pathways related to spermatogenesis and testicular maturation. In the 0.5- versus 2.5-year comparison, GO analysis revealed significant enrichment of reproductive and germ cell-related processes, including reproductive process (GO:0022414), spermatogenesis (GO:0007283), sperm motility (GO:0030317), cilium organization (GO:0044782), and microtubule-based movement (GO:0007018) (Figure 4A). KEGG analysis identified focal adhesion (adjusted *p* = 1.28 × 10^−7^) and ECM–receptor interaction (adjusted *p* = 8.08 × 10^−7^) as the most significantly enriched pathways (Figure 4B). These findings suggest extensive extracellular matrix remodeling and cell adhesion dynamics during the pubertal transition. Similar enrichment patterns were observed in the 0.5- versus 4.5-year comparison, whereas considerably fewer enriched GO terms and KEGG pathways were identified in the 2.5- versus 4.5-year comparison. To further dissect the molecular basis of this ECM remodeling, we examined the expression profiles of 60 key ECM-related genes across the three developmental stages (Figure 5). The heatmap reveals a coordinated down-regulation of ECM-related genes from prepuberty to puberty, including collagens (e.g., *COL4A1*, *COL4A2*, *COL6A1*, *COL6A2*), *laminins* (*LAMA1*, *LAMB1*, *LAMC1*), *integrins* (*ITGAV*, *ITGB5*, *ITGA9*), matrix metalloproteinases (*MMPs*; *MMP2*, *MMP14*), and tissue inhibitors of metalloproteinases (*TIMPs*; *TIMP1*, *TIMP2*, *TIMP3*), indicating coordinated changes in ECM components during the pubertal transition.

### 3.5. WGCNA Identifies Age-Associated Co-Expression Modules

**Figure 6:**
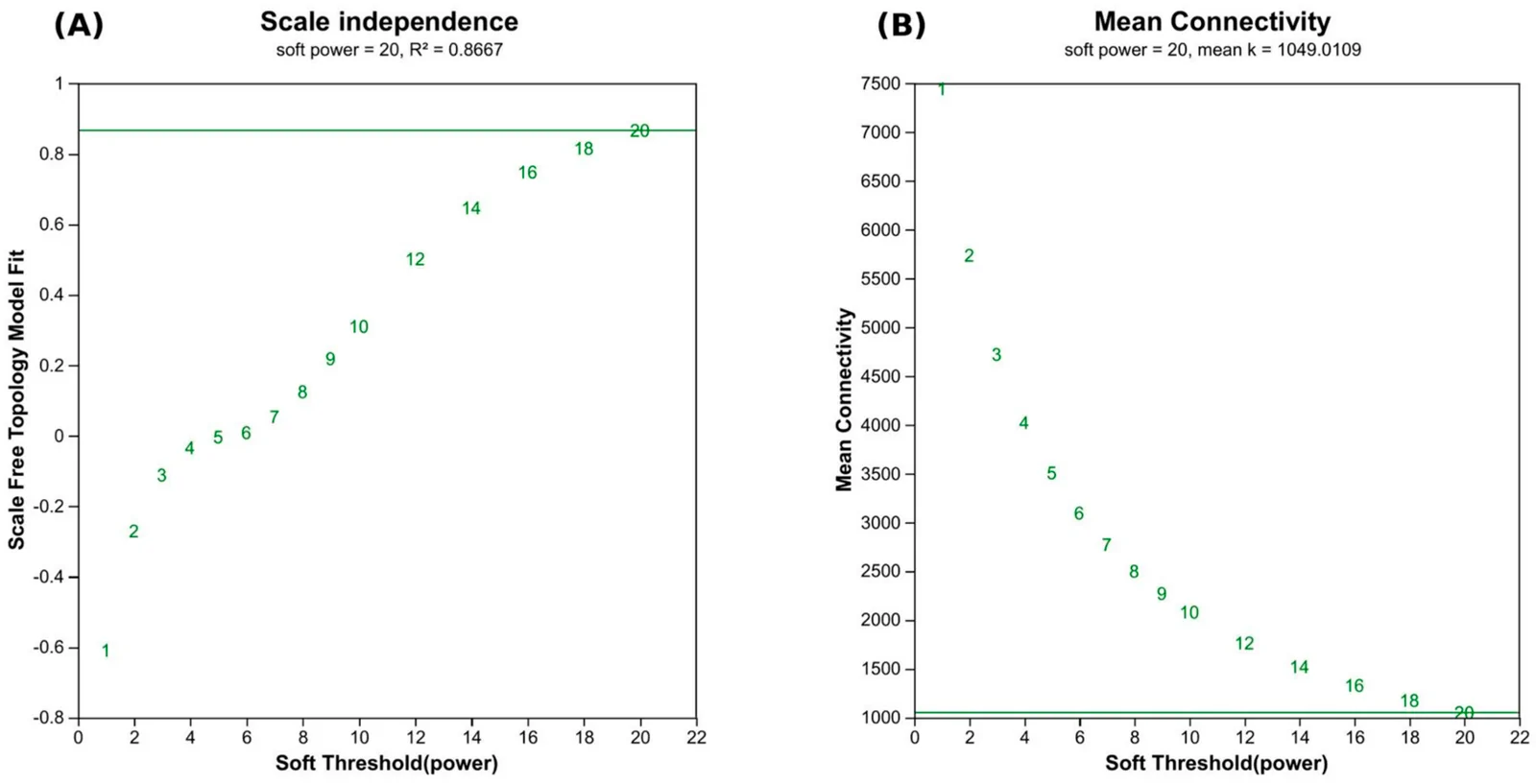
Selection of the soft-thresholding power for WGCNA. (**A**) Scale-free topology fit index (R^2^) as a function of soft-thresholding power (β). The green horizontal line indicates R^2^ = 0.8667. (**B**) Mean connectivity across different soft-thresholding powers. The green horizontal line indicates the mean connectivity at the selected soft-thresholding power (β = 20). A value of β = 20 was selected for network construction.

**Figure 7:**
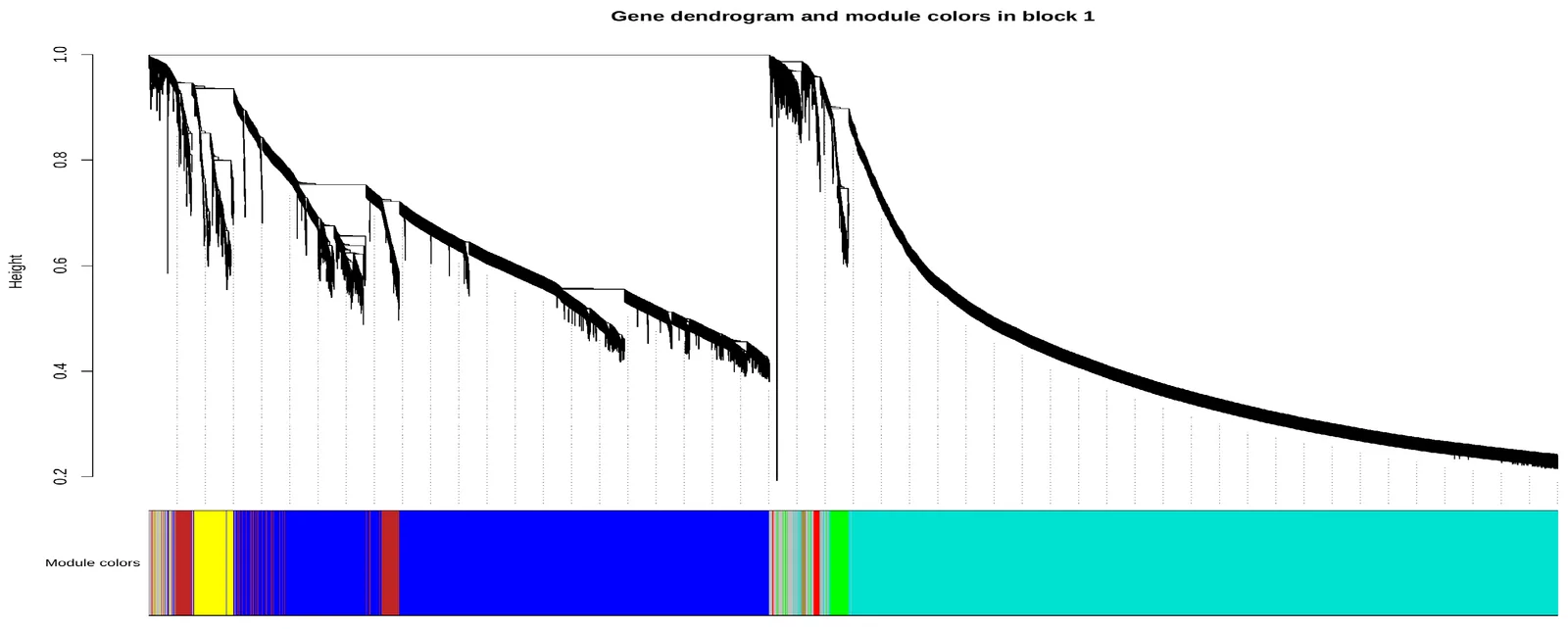
Hierarchical clustering dendrogram and module assignment generated by WGCNA. The upper panel shows the gene dendrogram based on topological overlap, and the lower color bar indicates module membership. A total of seven co-expression modules were identified.

WGCNA was performed using the 11,602 genes that passed low-expression filtering across all samples. The optimal soft-thresholding power was determined to be β = 20, at which the scale-free topology fit index reached R^2^ = 0.8667 (Figure 6). This value is higher than typically reported in similar studies (commonly β = 6–12); a sensitivity analysis across β = 6–20 showed that the module assignment of the core hub genes (*SPATA6*, *SPMIP11*, *TNP1*, and *ODF1*) was robust to the choice of β (Table S2). Hierarchical clustering based on the topological overlap matrix identified seven distinct co-expression modules, ranging in size from 67 to 5883 genes (Figure 7).

**Figure 8:**
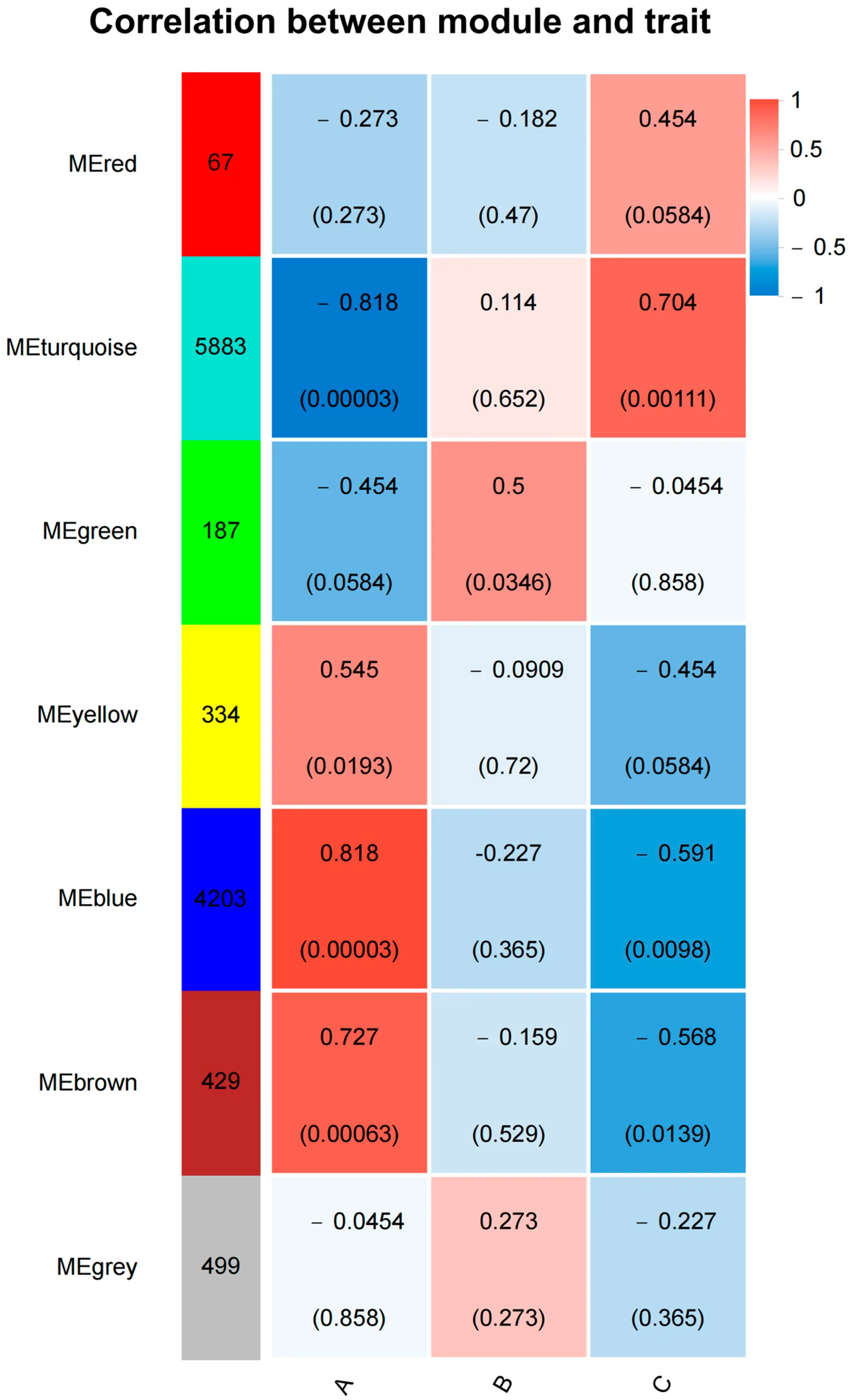
Module–trait relationship heatmap showing Pearson correlations between module eigengenes and the three developmental groups (0.5, 2.5, and 4.5 years). A, B, and C denote the 0.5-, 2.5-, and 4.5-year groups, respectively.

WGCNA identified seven co-expression modules whose eigengenes were correlated with developmental age (Figure 8). After Bonferroni correction across 21 tests (7 modules × 3 groups; adjusted threshold *p* < 0.0024), the turquoise module showed the strongest negative correlation with the 0.5-year group (r = −0.818, *p* = 3.0 × 10^−5^) and the strongest positive correlation with the 4.5-year group (r = 0.704, *p* = 1.1 × 10^−3^), whereas the blue module showed the opposite pattern (r = 0.818 with the 0.5-year group; r = −0.591 with the 4.5-year group), together indicating that the turquoise module is up-regulated and the blue module is down-regulated with developmental age. The remaining modules (red, green, yellow, brown, and grey) showed weaker or non-significant correlations with developmental age. The turquoise module was enriched for genes related to spermatogenesis, sperm flagellum assembly, fertilization, and energy metabolism, whereas the yellow, blue, and brown modules were enriched for genes involved in RNA processing and transcriptional regulation.

### 3.6. Key Hub Genes Associated with Testicular Maturation

In the turquoise module, which exhibited the strongest positive correlation with age and contained the largest number of genes (5883 genes), the top hub genes (|kME| > 0.99) included *SPATA6* (kME = 0.997) [42], *SPMIP11* (kME = 0.997) [43], *TNP1* (kME = 0.990) [44], *ODF1* (kME = 0.999) [45], and *PGK2* (kME = 0.999) [46]. These genes are involved in sperm head–tail junction assembly, flagellar microtubule complex formation, histone-to-protamine transition, and outer dense fibre formation, reflecting their roles in spermatid differentiation and maturation. The yellow module (334 genes) was negatively correlated with age and was enriched for genes involved in signal transduction and transcriptional regulation.

### 3.7. RT-qPCR Confirms Expression Patterns of Candidate Genes

RT-qPCR was performed to validate the expression patterns of six candidate DEGs identified by RNA-seq (*SPATA6*, *TNP1*, *COL4A1*, *COL6A1*, *FN1*, and *ITGAV*) across the three developmental stages. One sample (A-T-4) was excluded from the analysis due to suboptimal RNA quality (A260/A280 ratio = 1.64). The remaining 17 samples produced high-quality amplification, with melt curve analysis confirming single-product amplification for all six primer pairs.

One-way ANOVA revealed significant differences across developmental stages for *SPATA6* (F = 9.77, *p* = 0.0022), *TNP1* (F = 7.25, *p* = 0.0069), and *COL4A1* (F = 9.46, *p* = 0.0025), whereas *COL6A1*, *FN1*, and *ITGAV* showed no significant differences (*p* > 0.05). Tukey HSD post hoc analysis indicated that the expression of *SPATA6* and *TNP1* was significantly up-regulated between 0.5 and 2.5 years as well as between 0.5 and 4.5 years (*p* < 0.001), whereas *COL4A1* was significantly down-regulated across the same comparisons (*p* < 0.001). No significant differences were detected between the 2.5- and 4.5-year groups for any of the three significantly regulated genes, consistent with the RNA-seq findings of only 126 DEGs between these stages.

**Figure 9:**
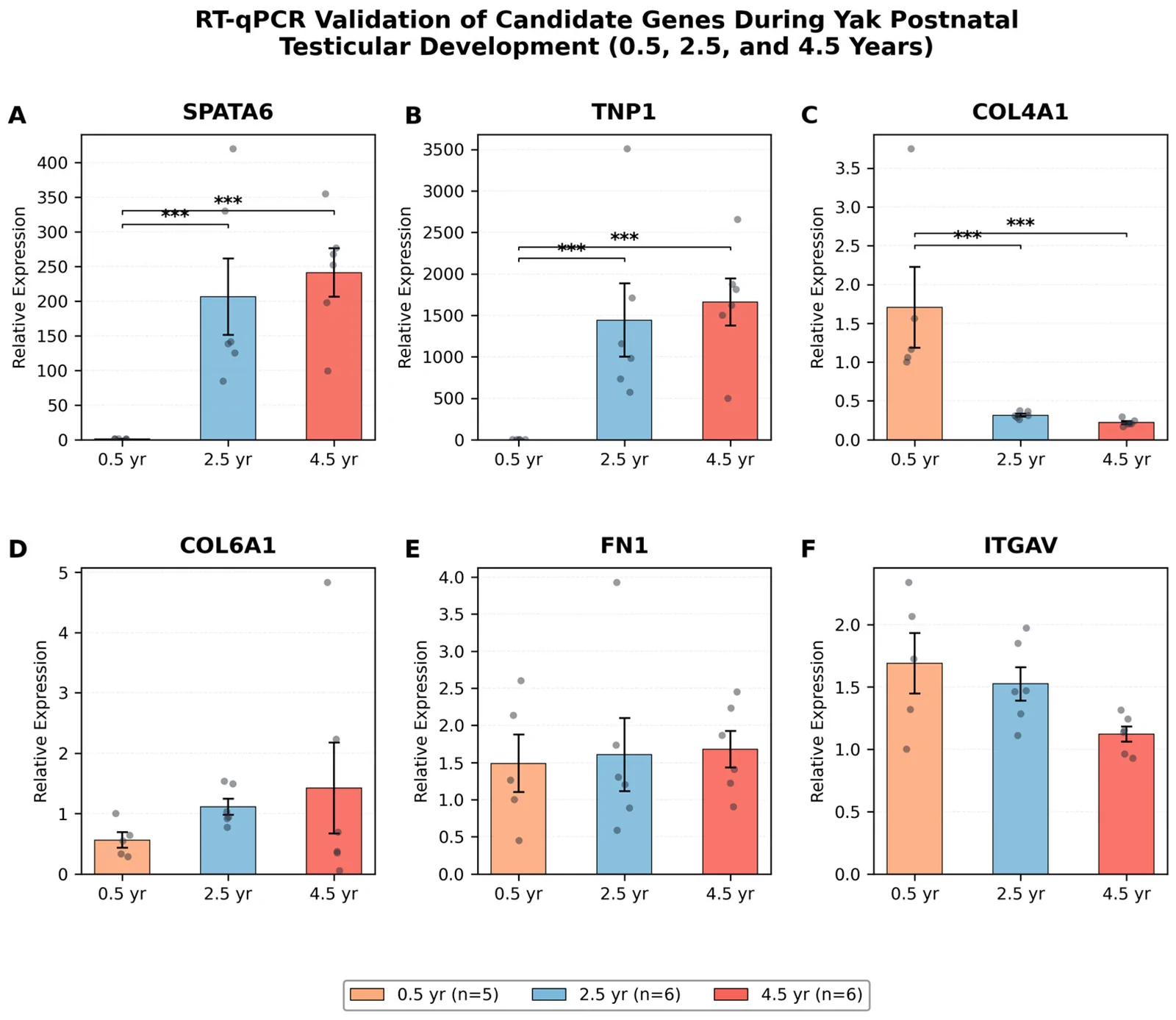
RT-qPCR validation of six candidate DEGs during yak postnatal testicular development. Bar charts show mean ± SEM relative expression (2^−ΔΔCt^) for *SPATA6*, *TNP1*, *COL4A1*, *COL6A1*, *FN1*, and *ITGAV* across three developmental stages: 0.5 yr (A, *n* = 5), 2.5 yr (B, *n* = 6), and 4.5 yr (C, *n* = 6). Statistical analysis was performed using one-way ANOVA with Tukey HSD post hoc test, *** *p* < 0.001. A-T-4 was excluded due to suboptimal RNA quality.

The RT-qPCR log_2_ fold changes (B vs. A) showed moderate concordance with RNA-seq values (Pearson r = 0.991, *p* < 0.001; Spearman ρ = 0.771), supporting the RNA-seq expression patterns for a subset of the validated genes. The qPCR validation results are presented in Figure 9, and detailed statistical parameters are provided in Table S6.

## 4. Discussion

This study provides a transcriptomic characterization of yak testicular maturation across three postnatal developmental stages using six biological replicates per group from a single breed. Previous transcriptomic studies have characterized mRNAs, lncRNAs, circRNAs, and microRNAs during yak testicular development using differential expression and ceRNA network analyses [23,24,25,26], although these studies examined a single developmental transition [28] or included animals from multiple breeds [28,29]. Our WGCNA-based approach complements these studies by providing a network-level view of developmental stage-associated co-expression modules and hub genes. The present design enabled the detection of both major transcriptional changes and more subtle age-associated differences. By integrating differential expression analysis with WGCNA, we moved beyond individual gene-level changes to identify co-expression modules and hub genes associated with testicular maturation.

The histological findings demonstrated clear developmental milestones. At 0.5 years, seminiferous tubules were narrow and contained Sertoli cells and spermatogonia but no spermatids. By 2.5 years, the complete spermatogenic lineage was present, including elongating spermatids. At 4.5 years, the testes exhibited fully mature morphology with abundant luminal spermatozoa. These observations are consistent with previous reports of yak testicular development [28] and with the pubertal progression described in cattle [13,16] and sheep [17,18].

The transcriptomic data revealed a marked asymmetry in DEG distribution, with 9646 DEGs identified between 0.5 and 2.5 years but only 126 DEGs between 2.5 and 4.5 years. This approximately 77-fold difference in DEG count suggests that the transcriptional program underlying spermatogenesis is largely established during puberty and subsequently remains relatively stable, requiring only limited adjustments to maintain adult testicular function. Similar patterns have been reported in cattle [13] and sheep [17,18], suggesting a conserved developmental feature among ruminants.

The identities of the DEGs further support this interpretation. Genes up-regulated at 2.5 and 4.5 years were enriched for functions related to spermatogenesis, sperm motility, and cilium organization, processes characteristic of meiotic and post-meiotic spermatogenesis [10,11,17]. Notably, focal adhesion (*p* = 1.28 × 10^−7^) and ECM–receptor interaction (*p* = 8.08 × 10^−7^) were among the most significantly enriched pathways during the pubertal transition. Beyond their established roles in cell–substrate adhesion, these pathways have been implicated in multiple aspects of testicular development and function. We propose three interconnected, non-mutually exclusive mechanisms through which ECM remodeling and focal adhesion signaling may contribute to yak testicular maturation during puberty.

First, the BTB [31,47,48,49], which is formed by tight junctions, adherens junctions, and gap junctions between Sertoli cells, is established during puberty in most mammals [31]. A limitation of the present study is that bulk RNA-seq cannot determine the cellular origin of the enriched ECM- and focal adhesion-related transcripts, which may be expressed by Sertoli cells, peritubular myoid cells, or other somatic cell populations. The BTB creates the immune-privileged adluminal compartment required for meiotic and post-meiotic germ cell development. ECM components, including laminins [32], collagens, and fibronectin, interact with integrin receptors on Sertoli cells. These interactions regulate the phosphorylation and organization of tight junction proteins such as occludin, claudin-11, and ZO-1, thereby controlling BTB assembly and remodeling [31,49]. Focal adhesion kinase (FAK) [48], a key mediator of integrin signaling, is essential for maintaining BTB integrity. Disruption of FAK activity in Sertoli cells compromises the tight junction permeability barrier, providing direct evidence of functional coupling between ECM-derived signals and BTB dynamics [18,32]. In this context, the enrichment of ECM–receptor interaction and focal adhesion pathways during the transition from 0.5 to 2.5 years is consistent with the activation of molecular programs associated with BTB establishment during puberty.

Second, germ cells migrate from the basal compartment to the adluminal compartment as they progress from spermatogonia to spermatocytes and ultimately spermatids. This movement is thought to require continuous remodeling of adhesive interactions at the Sertoli cell–germ cell interface [31,32]. FAK and integrin-linked kinase (ILK), two central components of focal adhesion signaling, transmit ECM-derived signals to the actin cytoskeleton and may coordinate the structural changes required for germ cell translocation. The presence of focal adhesion and actin cytoskeleton-related genes among the DEGs further supports this interpretation and suggests coordinated regulation of ECM signaling and cytoskeletal organization during pubertal spermatogenesis.

Third, the substantial increase in seminiferous tubule diameter observed between 0.5 and 2.5 years (Figure 1) is likely accompanied by extensive remodeling of the tubular basement membrane, a structure composed primarily of ECM proteins. This process involves both degradation of pre-existing matrix components by matrix metalloproteinases and synthesis of new ECM proteins by Sertoli cells and peritubular myoid cells. The upregulation of ECM-related genes observed during this developmental interval is therefore consistent with active remodeling of the seminiferous tubule microenvironment. Such remodeling may be required to accommodate tubular expansion and support the increasing spermatogenic output associated with puberty. Similar ECM-related transcriptional signatures have been reported during pubertal testicular development in sheep [18,21] and cattle–yak hybrids [22,30], suggesting that ECM remodeling represents a conserved feature of testicular maturation across bovids. Consistent with this, comparative metabolomic profiling in yellow cattle has implicated metabolic pathways linked to ECM signaling in testis biology [50].

WGCNA provided complementary systems-level insights into the molecular programs underlying testicular maturation. The seven co-expression modules showed varying degrees of association with developmental age, with four module-age correlations remaining significant after Bonferroni correction. The turquoise module, which contained 5883 genes and exhibited the strongest positive association with age, was enriched for genes associated with spermatid differentiation, maturation, and flagellum assembly. Notably, the hub genes of this module included *SPATA6*, which is required for sperm head–tail junction assembly [42]; *SPMIP11*, a component of the sperm flagellar microtubule inner protein complex [43]; *TNP1*, which mediates the histone-to-protamine transition during spermiogenesis [44]; *ODF1*, a major structural component of sperm outer dense fibres [45]; and *PGK2* [46]. The coordinated expression of these genes within a single module highlights the highly integrated nature of terminal spermatid differentiation and suggests their potential roles in male germ cell maturation in yak. The yellow, blue, and brown modules were negatively correlated with age and enriched for genes associated with signal transduction and transcriptional regulation, contrasting with the spermatogenesis-related turquoise module.

Several limitations should be considered when interpreting these findings. First, the analysis was conducted using bulk testicular tissue, which represents a composite transcriptome derived from multiple cell populations, including germ cells at different developmental stages, Sertoli cells, Leydig cells, peritubular myoid cells, and vascular cells. Consequently, part of the observed transcriptomic changes during puberty may reflect shifts in cell-type composition, and the cellular origins of the identified transcriptional signatures and hub genes cannot be determined directly. Single-cell RNA sequencing would provide the resolution required to define cell-type-specific transcriptional programs and assign hub genes to their cellular sources [22,51]. Second, the present analyses are inherently correlative. Although differential expression and co-expression approaches effectively identify candidate genes and pathways, experimental validation at the protein and functional levels remains necessary. Techniques such as immunohistochemistry, Western blotting, and targeted gene perturbation would help establish the biological roles of the identified candidates. Third, the histological characterization was qualitative rather than quantitative, as these testicular samples are currently being subjected to proteomic and metabolomic analyses to preserve sample integrity; future morphometric measurements (e.g., seminiferous tubule diameter and epithelial height) will strengthen the link between phenotypic maturation and transcriptomic changes. Fourth, all samples were collected during the cold season. Given the pronounced seasonal reproductive characteristics of yak [6], the influence of seasonal variation on testicular gene expression warrants further investigation.

Fifth, RT-qPCR validation was performed on a subset of six DEGs using the same RNA samples as the RNA-seq experiment; the strong correlation between qPCR and RNA-seq log_2_ fold changes (Pearson r = 0.991, *p* < 0.001) indicates agreement between the two methods for the tested genes, whereas the lower Spearman rank correlation (ρ = 0.771) reflects the disproportionate influence of genes with large fold changes (particularly *SPATA6* and *TNP1*) on the rank-based metric, a pattern consistent with the wider dynamic range of RNA-seq for highly differentially expressed genes; however, the limited number of validated genes precludes comprehensive assessment of the RNA-seq quantitative accuracy. Sixth, three ECM-related genes (*COL6A1*, *FN1*, and *ITGAV*) showed differential expression in the RNA-seq analysis but did not reach statistical significance in the RT-qPCR validation. This discrepancy may reflect relatively modest fold changes in the RNA-seq data for these genes, differences in transcript isoform detection between the two platforms, or greater biological variability in ECM gene expression across individuals. Further validation using additional biological replicates or protein-level assays would help clarify the role of these ECM components in yak testicular development.

Several avenues for future research emerge from this work. Single-cell transcriptomic analyses across developmental stages would clarify the contributions of individual testicular cell populations to the observed transcriptional changes. Proteomic profiling of the same tissue samples is planned and will enable integrated transcriptomic–proteomic analyses to determine whether the molecular signatures identified here are reflected at the protein level. Comparative analyses between yak and cattle, two closely related species adapted to markedly different environments, may reveal lineage-specific regulatory mechanisms governing testicular development and reproductive seasonality. Further functional investigation of ECM-associated genes, particularly FAK and its downstream signaling pathways, may help elucidate the molecular mechanisms linking extracellular matrix remodeling to BTB dynamics during puberty. Finally, integrating transcriptomic and proteomic data with endocrine measurements, including testosterone [51], follicle-stimulating hormone (FSH), and luteinizing hormone (LH), would provide a more comprehensive understanding of the endocrine and molecular networks that regulate testicular maturation in yak [6].

## 5. Conclusions

This study provides a comprehensive characterization of testicular maturation in the Tianzhu white yak through the integration of histology, RNA-seq, differential expression analysis, and WGCNA. The results indicate that the most extensive molecular changes occur during the pubertal transition between 0.5 and 2.5 years of age, whereas the testicular transcriptome undergoes relatively few changes from puberty to adulthood. The prominent enrichment of focal adhesion and ECM–receptor interaction pathways suggests that ECM remodeling may play an important role in testicular maturation by supporting BTB establishment, germ cell development, and seminiferous tubule expansion. WGCNA identified seven age-associated co-expression modules and highlighted hub genes, including *SPATA6*, *SPMIP11*, *TNP1*, and *ODF1*, that are associated with spermatid differentiation and maturation. Together, these findings provide new insights into the molecular mechanisms underlying yak testicular development and establish a foundation for future functional and multi-omics studies of reproduction in this economically important high-altitude species.

## Figures and Tables

**Table 1:** Summary of RNA sequencing data and mapping statistics.

Group	Clean Reads (Million)	Q20 (%)	Q30 (%)	GC (%)	Total Mapped (%)	Unique Mapped (%)
0.5 yr (*n* = 6)	37.67 ± 3.06	99.21 ± 0.01	96.20 ± 0.04	47.57 ± 0.24	92.77 ± 0.30	80.07 ± 0.42
2.5 yr (*n* = 6)	39.37 ± 2.57	99.22 ± 0.02	96.19 ± 0.05	48.11 ± 0.31	93.22 ± 0.19	79.28 ± 0.29
4.5 yr (*n* = 6)	40.09 ± 4.38	99.22 ± 0.01	96.20 ± 0.03	48.52 ± 0.25	93.36 ± 0.24	79.02 ± 0.25

Values are presented as mean ± SD (*n* = 6 per group). Q20 and Q30 denote the percentages of bases with Phred quality scores ≥20 and ≥30, respectively. GC, guanine–cytosine content.

**Table 2:** Numbers of differentially expressed genes identified in pairwise comparisons among the three developmental stages of yak testicular development.

Comparison	Up-Regulated	Down-Regulated	Total DEGs
0.5 yr vs. 2.5 yr	5249	4397	9646
0.5 yr vs. 4.5 yr	5578	4796	10,374
2.5 yr vs. 4.5 yr	60	66	126

DEGs were identified using DESeq2 with thresholds of |log_2_FC| > 1 and adjusted *p* < 0.05 (Benjamini–Hochberg correction). Up- and down-regulated genes are reported relative to the younger developmental stage in each comparison (0.5 yr as the baseline for the first two comparisons and 2.5 yr for the third). In the raw output from the Majorbio Cloud Platform, up- and down-regulation are defined relative to the first-named group in each comparison label; the values presented here were re-anchored for biological interpretability.

## Data Availability

The RNA-seq data have been deposited in the NCBI Sequence Read Archive (SRA) under BioProject accession number PRJNA1479487 (sample accessions SAMN61084578–SAMN61084595) and will be made publicly available upon acceptance of the manuscript.

## Supplementary Materials

The following supporting information can be downloaded at https://www.mdpi.com/article/10.3390/ani16182853/s1, Figure S1: Volcano plot of DEGs between 2.5-year and 4.5-year groups; Figure S2: GO enrichment analysis of DEGs between 2.5-year and 4.5-year groups; Figure S3: KEGG pathway enrichment analysis of DEGs between 2.5-year and 4.5-year groups; Figure S4: GO molecular function and cellular component enrichment; Figure S5: Correlation between RT-qPCR and RNA-seq log_2_ fold changes for six validated genes; Table S1: Complete list of differentially expressed genes identified across all pairwise comparisons; Table S2: WGCNA module assignments, module statistics, preprocessing summary for all analyzed genes, and sensitivity analysis of the soft-thresholding power (β = 6–20); Table S3: Complete GO enrichment analysis results for all pairwise comparisons; Table S4: Complete KEGG pathway enrichment analysis results for all pairwise comparisons; Table S5: Primer sequences used for RT-qPCR validation; Table S6: Statistical parameters of RT-qPCR validation (ANOVA and Tukey HSD results).

## Abbreviations

BTB	blood–testis barrier
DEG	differentially expressed gene
ECM	extracellular matrix
FAK	focal adhesion kinase
GO	Gene Ontology
H&E	hematoxylin and eosin
KEGG	Kyoto Encyclopedia of Genes and Genomes
kME	module membership
PCA	principal component analysis
RT-qPCR	reverse transcription quantitative polymerase chain reaction
TPM	transcripts per million
WGCNA	weighted gene co-expression network analysis
SRA	Sequence Read Archive
SEM	standard error of the mean
MMP	matrix metalloproteinase
TIMP	tissue inhibitor of metalloproteinases
ILK	integrin-linked kinase
